# Paediatric hypopituitarism: a case report and management challenges in a resource poor setting

**DOI:** 10.11604/pamj.2020.37.170.23656

**Published:** 2020-10-20

**Authors:** Isaac Oludare Oluwayemi, Oladele Simeon Olatunya, Ezra Olatunde Ogundare, Adebukola Bidemi Ajite, Adefunke Olarinre Babatola, Adewuyi Temidayo Adeniyi, Akinwumi Kolawole Komolafe

**Affiliations:** 1 Department of Paediatrics, Faculty of Clinical Sciences, College of Medicine, Ekiti State University, Ado-Ekiti, Ekiti State, Nigeria,; 2 Department of Paediatrics, Ekiti State University Teaching Hospital, Ado-Ekiti, Ekiti State, Nigeria

**Keywords:** Hypopituitarism, management, adolescent, developing country, challenges

## Abstract

Hypopituitarism, a deficiency of one or more of the hormones produced by the pituitary gland, is a rare disorder. It can be congenital or acquired. Case report on childhood hypopituitarism is rare in Nigeria. We present a 15-year-old boy, second of a set of twins, who presented with short stature and delayed puberty. Subtle difference in stature, was noticed on review of their childhood pictures by 2 years of age though disparity in stature became obvious to the parents at 6 years of age and it became embarrassing at 15 years of age when parents decided to seek medical attention. He was a product of term gestation with birth weight of 3.2kg; there was no history suggestive of birth trauma. Developmental milestone in the first two years of life was essentially normal like his unaffected twin brother. At presentation both height and weight were below 3^rd^ percentile for age, he had a low blood pressure of 80/50mmHg, infantile male external genitalia with testicular volume of 2ml, bone age of 7 years, very low serum testosterone, growth hormone, adrenocorticotropic hormone, thyroxine, follicle stimulating hormone, leutenizing hormone, Cortisol and high thyroid stimulating hormone. He achieved remarkable improvement in physical activity, height, weight and hormonal profile within the first 7 months of hormone replacement therapy but could not sustain therapy because of financial constraint. Paediatric hypopituitarism is a rare and treatable disorder. Early presentation, diagnosis and appropriate hormone replacement therapy at affordable price is essential for survival and good prognosis.

## Introduction

Hypopituitarism occurs when there is deficiency of one or more of the eight hormones produced by the pituitary gland [1]. Hypopituitarism is a rare disorder but it is likely to be underdiagnosed in people with traumatic brain injury [1]. The incidence in children is possibly fewer than 3 cases per million people per year [2]. The prevalence of hypopituitarism in children is not well documented; there are only pockets of case reports and case series in literature [3, 4]. Hypopituitarism can be congenital or acquired, hence it can occur in neonates, infants, children, adolescents and adults [2]. Any disease that affects the pituitary gland, the stalk of pituitary gland or the hypothalamus can cause hypopituitarism. Reports on paediatric hypopituitarism are very scarce. There is no known report from our study locality hence, this case report to create awareness and ensure high index of suspicion which will lead to early diagnosis and management of affected children.

## Patient and observation

**Figure 1:**
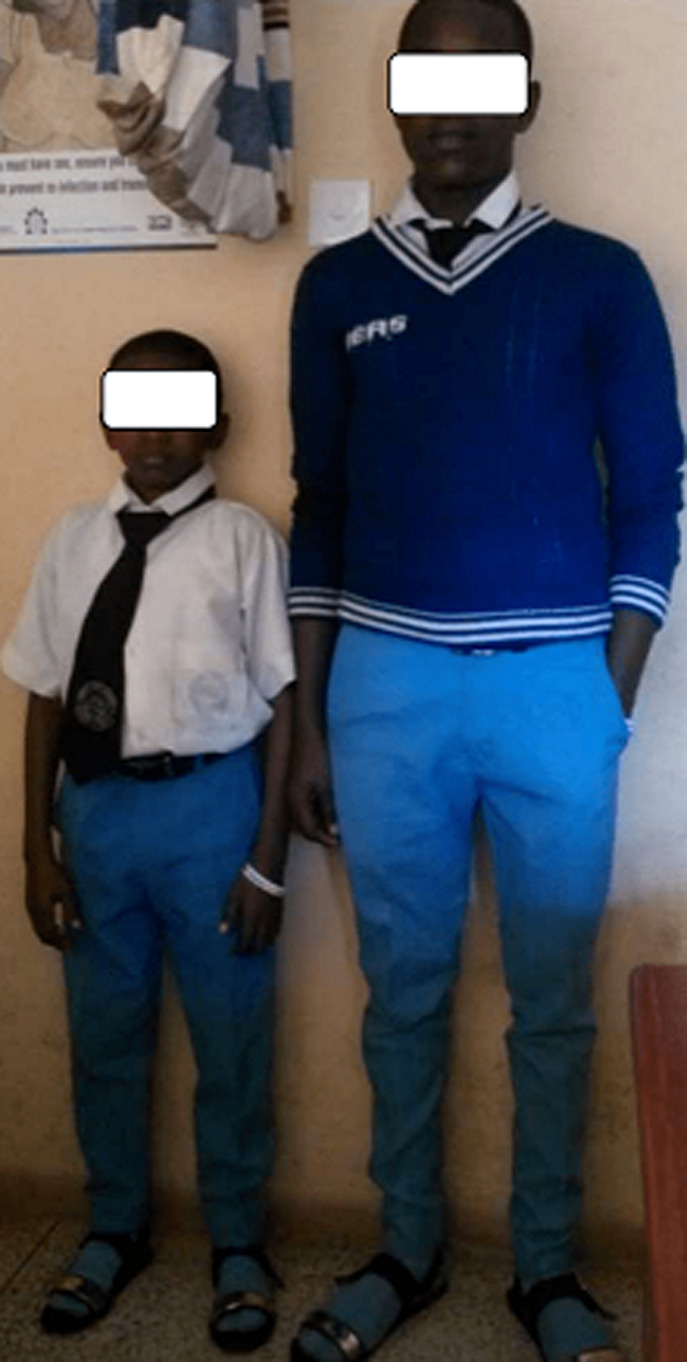
combined hormone deficiency patient (left) and his normal twin brother

KA presented at our clinic at the age of 15 years with short stature and delayed puberty. Parents started noticing obvious difference in their stature by 6 years of age but didn´t give it a serious thought until he was 15 years old when the difference in their stature became embarrassing. This prompted the parent to take him to a secondary health facility from where he was referred to our facility. A closer review of the pictures taken with his twin brother showed that the difference in stature became noticeable at the age of 2 years and became very glaring from age 6 years upward. He is the second of a set of twin (both male), product of term gestation delivered via spontaneous vaginal delivery. He cried immediately after birth and had normal birth weight of 3.2kg. Neonatal period was uneventful. He had normal development in the first year of life like his twin brother. He is the 5^th^ child in a monogamous family setting and all his elder siblings (3 males and 1 female) are of normal stature for age. At presentation, he was obviously small for age (Figure 1), had small face, thin voice, not in any distress, he was short statured with height of 131.5cm (< 3^rd^ percentile), weight was 25kg (< 3^rd^ percentile). Arm span was 138cm and arm span to height ratio was 1:1.05. He had a low blood pressure of 80/50mmHg and normal heart sounds. He had infantile prepubertal genitalia with stretched penile length of 4.5cm and testicular volume of 2ml. There was no pubic or axillary hair growth. He communicated intelligently though very shy, had a low occipitofrontal circumference of 54cm. His bone age was 7 years; haemoglobin genotype was AA; normal serum electrolyte, urea and creatinine; cranial computerized tomography (CT) scan was essentially normal. Hormonal profile showed very low serum testosterone, growth hormone, adrenocorticotropic hormone (ACTH), thyroxine, follicle stimulating hormone (FSH), luteinizing hormone (LH), fasting cortisol and high thyroid stimulating hormone (Table 1).

**Table 1:** serial results of hormonal profile

		Hormonal Levels	
Hormones	Reference range	At presentation	One year after hormone replacement therapy
FSH	1.0-14.0 mIU/ml	1.0	0.0
LH	0.7-7.4 mIU/ml	0.3	0.2
Testosterone	3.0-10 ng/ml	0.0	0.0
fT3	1.6-4.2 pg/ml	1.0	2.5
fT4	0.8-2.0 ng/dl	0.2	0.9
TSH	0.5-3.7μIU/ml	4.2	0.4
Growth hormone	5-8 ng/ml	0.07	0.5
Cortisol	7-28μg/dl	2.5	5.3

An assessment of panhypopituitarism was made and the patient was commenced on hormone replacement therapy with growth hormone therapy, Levothyroxine, and low dose prednisolone. Subcutaneous HCG was added at the 7^th^ month of hormone replacement therapy and FSH added at the 11^th^ month to induce spermatogenesis. He gained 2.5cm increase in height and 2kg increase in weight within the first 7 months of hormone replacement therapy with growth hormone, L-thyroxine and prednisolone. Testicular volume also increased to 3mls and there was significant improvement in physical activity. Repeat hormonal profile showed remarkable improvement in the serum hormonal levels (Table 1). However, supply of the hormones was erratic for the 2-year period of patient´s follow up at the clinic and thereafter stopped abruptly, because of financial constraint, before patient could get to start receiving testosterone which would have further improved his outlook. A month supply of hormones for replacement therapy costs about 2,500 USD which amounts to 30,000 USD annually. Efforts at securing assistance for patient from pharmaceutical companies within and outside the nation proved abortive. Patient was eventually lost to follow up after 2 years of poor treatment compliance. Consent of the patient´s father was secured to use patient´s clinical information to source for help for the patient before he stopped coming to clinic and he has been unreachable thereafter till the writing of this case report.

## Discussion

The pituitary gland is located at the base of the brain and is composed of the anterior and the posterior regions. The anterior region produces 6 of the 8 pituitary hormones (growth hormone (GH), adrenocorticotropic hormone (ACTH), thyroid stimulating hormone (TSH), luteinizing hormone (LH), follicle stimulating hormone (FSH) and prolactin) while the hormones of posterior region are antidiuretic hormones (ADH) and oxytocin [2].

Aetiology of hypopituitarism can be congenital or acquired. Congenital causes include: perinatal injuries (birth asphyxia, traumatic deliveries), interrupted pituitary stalk [5], absent or ectopic neurohypophysis, Pallister-Hall syndrome (hypothalamic hamartoma and polydactyly), genetic disorders [2, 6] (isolated GH deficiency, PIT1 and PROP1 mutations, septo-optic dysplasia, isolated gonadotropin deficiency) and developmental central nervous system defect (anencephaly, holoprosencephaly, pituitary aplasia or hypoplasia) [2]. Acquired causes of hypopituitarism include infiltrative disorders (tuberculosis, sarcoidosis, histiocytosis X, lymphocytic hypophysitis) and tumors [7, 8] (craniopharyngioma, germinoma, glioma, astrocytoma). The cause in the index patient is most likely congenital considering the early onset manifestation from the age of 2 years. There was no history suggestive of perinatal asphyxia or traumatic birth injury though that cannot be completely ruled out in twin gestation delivered per vaginam. The developmental milestone of the patient is comparable to that of his normal statured twin brother apart from his short stature and they were also in the same class educationally. Cranial CT was also reported as normal. There was no dysmorphic feature suggestive of Pallister-Hall syndrome. Other possible cause in the index patient is genetic mutation which is difficult to assess because of unavailability of investigative facilities for genetic studies.

Early diagnosis of hypopituitarism and appropriate hormone replacement therapy are essential for prevention of unnecessary morbidity and mortality. Clinical features of isolated or multiple deficiencies in pituitary hormones can include hypoglycaemia (weakness, headache, sweating, confusion, convulsion), adrenal crisis (profound hypotension, severe shock, death), short stature, hypogonadism (delayed puberty, infertility), osteoporosis (resulting in increased risk for fracture in adulthood) [2]. An important contributor to mortality in patient with hypopituitarism is growth hormone deficiency. Growth hormone deficiency of childhood onset has an increased hazard ratio of greater than 3.0 for morbidity [9].

The index patient presented at the age of 15 years with short stature and delayed puberty. The psychological trauma he would have gone through because of the embarrassing short stature is better imagined; more so that he often sees his twin brother growing well while his own stature was like that of a 7-year-old boy. This could have been avoided if presentation and diagnosis were made earlier and fund was readily available for appropriate hormone replacement therapy. Perhaps, the outcome could have been different for the patient if he was enrolled on National Health Insurance Scheme (NHIS) and the needed replacement hormones and laboratory tests were made available at very low cost to enhance early presentation, diagnosis, and treatment compliance. This will aid good therapeutic response, as shown in a previous study that parents of children with chronic diseases, who are not enrolled on NHIS, often incurred catastrophic health expenses [10]. The needed yearly 30,000 USD to purchase hormones for hormone replacement therapy is a great burden on the meager family income meant to take care of seven family members. Notwithstanding the erratic supply of hormones, the index patient experienced an encouraging increase in height, and weight within the first 7 months of growth hormone therapy but could not be sustained because of financial constraint. He did not have any clinical or biochemical feature of hypoglycaemia or adrenal crisis during the 2 years therapy at the Paediatric endocrinology clinic. This patient is likely to have responded well to hormone replacement therapy if there was regular supply of the appropriate hormones for replacement therapy.

## Conclusion

Paediatric hypopituitarism has multifactorial etiologies and can occur in neonates, infants, children and adolescents. Increased awareness, early diagnosis and appropriate hormone replacement therapy at affordable price is essential for survival and good prognosis. The cost of hormone replacement therapy is very high and beyond the reach of most patients in developing countries. Enrolling paediatric endocrine patients on NHIS and including the hormones on NHIS drug list will be a great panacea.
